# Serial dependence in estimates of the monetary value of coins

**DOI:** 10.1038/s41598-022-24236-z

**Published:** 2022-11-23

**Authors:** Yukihiro Morimoto, Shogo Makioka

**Affiliations:** 1 Department of Sustainable System Sciences, Osaka Prefecture University, 1-1, Gakuen-Cho, Naka-Ku, Sakai, Osaka 599-8531 Japan; 2 Department of Psychology, Osaka Metropolitan University, 1-1, Gakuen-Cho, Naka-Ku, Sakai, Osaka 599-8531 Japan

**Keywords:** Perception, Human behaviour

## Abstract

Perceptions of current stimuli are sometimes biased toward or away from past perceptions. This phenomenon is called serial dependence. However, it remains unclear whether serial dependence originates from lower-order perceptual processing, higher-order perceptual processing or cognitive processing. We examined the effects of serial dependence when participants estimated the total number of coins or the monetary value of coins displayed and found attractive effects in both tasks. The attractive effect observed in the value estimation task suggests that serial dependence occurs through higher-order cognitive processes during calculation. We also examined the effect of response history (i.e., the responses of participants on previous trials), with multiple regression analyses that simultaneously evaluated the effects of the previous stimuli and responses. In both number and value estimation tasks, the immediately prior response had an attractive effect on current responses, while the immediately prior stimuli exerted a repulsive effect. This pattern suggests that the attractive serial dependence found in the single regression analysis was due to the correlation between stimulus and response in the previous trials and that the effect of past stimuli per se may be an adaptation that increases sensitivity to current stimuli.

## Introduction

Past perceptual experiences inevitably influence current perceptions of the external world. Serial dependence is one such phenomenon in which the perception of a current stimulus is attracted to (or repelled from) the perception of a past stimulus^1,2^. When a response is induced by past experience, it is called attractive serial dependence, and when it is repelled, it is called repulsive serial dependence^3^. Repulsive serial dependence is a phenomenon equivalent to aftereffects that was studied before the discovery of serial dependence^4^. Unless otherwise specified, serial dependence refers to attractive serial dependence.

The nature of serial dependence has been examined in tasks using diverse types of stimuli, including tilt perception^2,5^, number perception^1,6,7^, motion perception^8^, face perception^9^, and sense of agency^10^. Attractive serial dependence is thought to stabilize the perception of continuously changing stimuli in noise, such as blinking or occlusion by objects^5,11^. Repulsive serial dependence increases sensitivity to change and facilitates adaptation to new situations. These two functions are thought to allow us to optimally perceive the external environment^3,12^. Various hypotheses have been proposed as mechanisms underlying this phenomenon, including a continuity field that supports visual stability and continuity^2^, the influence of memory and response biases^13^, feedback from higher-order processing^14^, and the effect of perceptual decision templates^3^.

The question of whether serial dependence originates from lower-order perceptual processing, higher-order perceptual processing or cognitive processing remains unanswered. Findings in favor of lower-order processing include the spatial localization of serial dependence^2,15,16^, while those in favor of higher-order processing include the attention requirements of serial dependence, that serial dependence can be induced by different stimuli representing the same attribute^14,17^, that it requires conscious perception^18,19^, and that it can be explained as a phenomenon of the decision-making process rather than the effect of a past stimulus^3^. It has been reported that the proximity (similarity) between the stimulus to be responded to and the preceding stimulus in the feature space affects serial dependence^13,20,21^. Fritsche et al. examined the effects of attention and feature proximity on serial dependence by conducting a visual search task followed by a test task in which participants adjusted the tilt of a line. Targets in the visual search task caused positive serial dependence when their orientations were similar to that of the test stimulus. Distractors caused attractive serial dependence when their orientations were close to that of the test stimulus but produced repulsive serial dependence when their orientations were far apart. These findings suggest that feature-based attention may affect serial dependence, and it has also been reported that stimuli that should be actively ignored cause negative serial dependence^22^.

**Figure 1:**
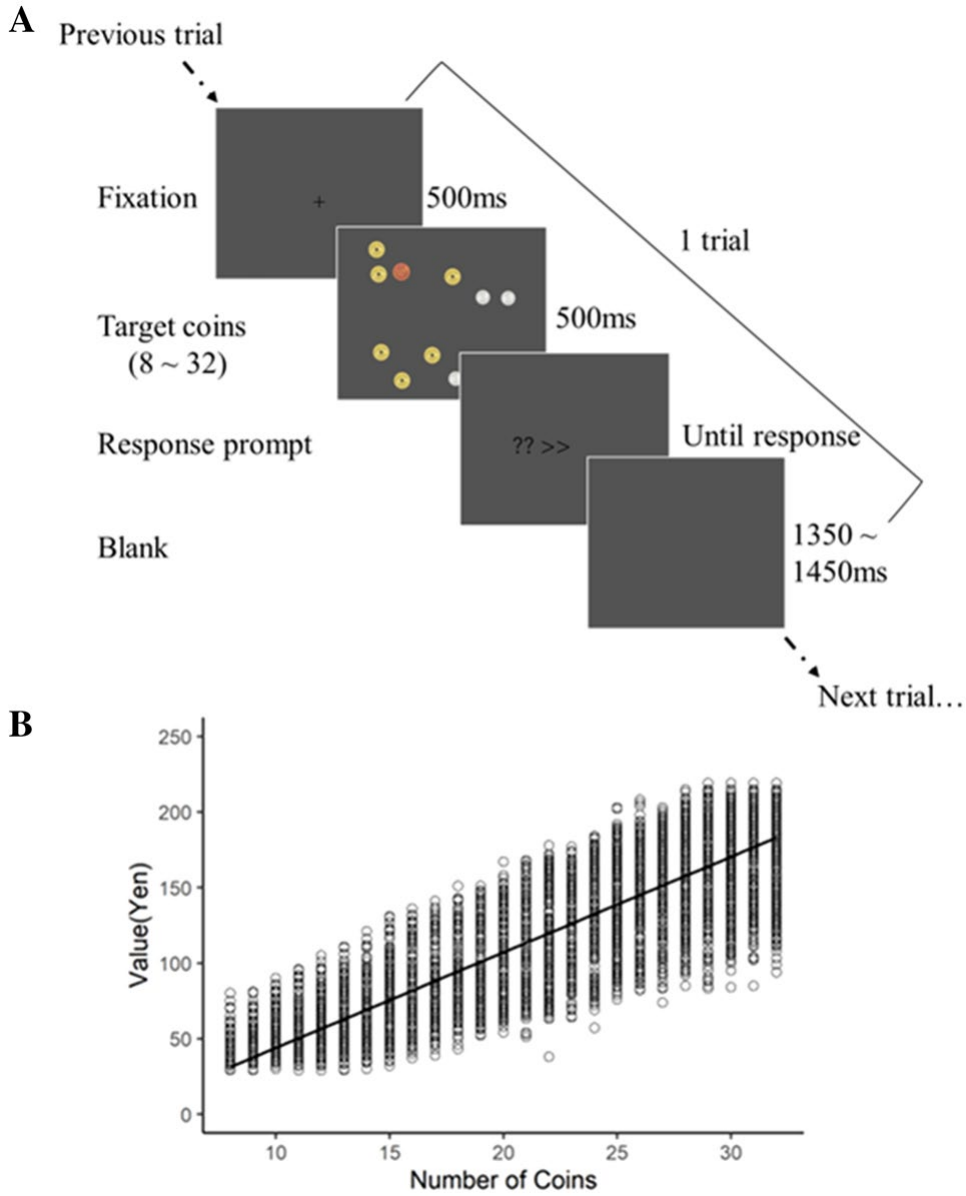
Experimental procedure and the distribution of the number and value of stimulus coins. (**A**) The stimulus presentation procedure in Experiments 1 and 2. On each trial, a fixation point was first presented for 500 ms, followed by the stimulus for 500 ms. The target stimuli included three types of coins, and the total number of coins ranged from 8 to 32. A silver coin represents one yen, a gold coin represents five yen, and a copper coin represents ten yen. A response prompt was then presented. Intertrial intervals varied randomly between 1350 and 1450 ms. (**B**) The distribution of the number and value of the stimulus coins in Experiment 2. The x-axis represents the number of coins displayed, and the y-axis represents the monetary value of coins displayed.

Previous studies on the serial dependence of numbers have used a task involving arrays with various numbers of dots^1,6,16^. In the present study, we aimed to examine the level of processing underlying serial dependence by using a task that requires higher-order processing. The ability to perceive the number of objects is advantageous for survival; indeed, many animals can perceive numbers^23^. Humans, however, often use numbers that deviate from the number of objects. One such example is money. We examined serial dependence in two types of numbers: the number of coins displayed (number of objects) and the monetary value displayed (which differs from the number of objects). Using coins allowed us to investigate these two types of numbers using the same stimuli (Fig. 1).

The number of dots in a stimulus array is assumed to be estimated by a mechanism specific to number perception^24^ that automatically extracts number information from the stimulus. For stimuli consisting of coins, this perceptual mechanism would extract the number of coins. However, the task involving calculation of the monetary value displayed requires a higher-order process (i.e., estimating the number of coins of each type and adding up the amounts). Thus, by independently manipulating the amount and the number of coins, we investigated whether serial dependence was caused by the amount or number of coins.

The serial dependence in estimates of the number of dots is dependent on stimulus location, and the magnitude of the serial dependence effect remains constant between monocular and binocular viewing conditions^14,15,25^. These findings suggest that serial dependence in number perception occurs during relatively early processing in the visual cortex.

Additionally, serial dependence disappears and transforms into adaptation in experiments that use backward masking, which interferes with visual feedback processing^25^. Serial dependence was reported to occur between different types of stimuli, such as the number of dots and the number of screen flashes^14^ or the number of dots and the number of digits^17^. These findings suggest that both lower-order visual processing (with spatial localization) and higher-order processing (with abstract numbers) influence the serial dependence effect in number perception.

In contrast, in a task where participants adjust the tilt of the Gabor patch, the effect of serial dependence was due to responses to past stimuli rather than the tilts of the past stimuli^3^. Participant responses inevitably correlate with the tilts of the current stimuli. Thus, if the tilts of the stimuli affect serial dependence, participant responses to the stimuli will also affect serial dependence. However, if past responses have a stronger effect on serial dependence than past stimuli, the responses themselves are considered to affect serial dependence. Indeed, the influence of past responses was found to be greater than the influence of past stimuli in the serial dependence of tilt perception. Pascucci et al. argue that serial dependence is influenced by perceptual decisions regarding stimuli, not the stimuli themselves^3^, and is thus related to decision inertia^26^. On the other hand, serial dependence has been observed after trials in which participants did not respond. This suggests that attractive serial dependence cannot be explained solely by response history^2,27^. Studies on the perception of the number of dots have not previously examined the effect of past responses on serial dependence. We therefore examined the effect of past responses on the serial dependence of the number and monetary value of coins.

The first aim of this study was to confirm whether serial dependence influences the estimation of the monetary value of coins. If serial dependence is observed in the estimation of the monetary value from coins of various types, this finding would suggest that serial dependence occurs in higher-order processing in which calculation takes place. The second aim was to compare the effect of past stimuli with the effect of past responses on the two tasks. This comparison is expected to provide deeper insight into the mechanism by which serial dependence arises.

The yen coins used in Japan do not have a hierarchical structure of naming, where different names are assigned on the value of the coins (e.g., dollar and cent, or euro and cent). Six types of coins (1, 5, 10, 50, 100, and 500 yen) are widely circulated in contemporary Japan. The relationship between the denominations of each type is limited to 2 × , 5 × , and 10 × the previous type. These attributes make estimating the monetary value of coins easier than in other countries.

## Statistical analysis

In Experiment 1, participants were asked to estimate the total number of coins displayed. The serial dependence effect was analyzed by the following two approaches. First, following Fornaciai and Park^16^, a single regression analysis was conducted to obtain regression coefficients for each participant with the estimated error (response—true stimulus value) on the current trial as the objective variable and the past stimuli and past responses as explanatory variables. To compare the effects of past stimuli and past responses, we then conducted a multiple regression analysis in which all explanatory variables significant in the single regression analyses were included, and the estimated error (response—true stimulus value) was the objective variable. The means of the regression coefficients across participants on the single and multiple regressions were considered the serial dependence effects, in accordance with a previous study^16^. The significance of the serial dependence effects in both Experiments 1 and 2 was assessed by a one-sample *t* test against the null hypothesis that the mean value of the regression coefficients did not differ from zero. The false discovery rate (FDR)^28^ was calculated to control for the probability of type I errors given multiple comparisons; the FDR-corrected significance level was set at 5% in all analyses.

## Results

### Experiment 1

Participants were asked to estimate the total number of coins presented on the screen. The stimulus duration was 500 ms, and the number of coins ranged from 8 to 32. This made it impossible for participants to accurately count the number of coins. Participants thus answered by estimating the number of coins.

#### Task performance

**Figure 2:**
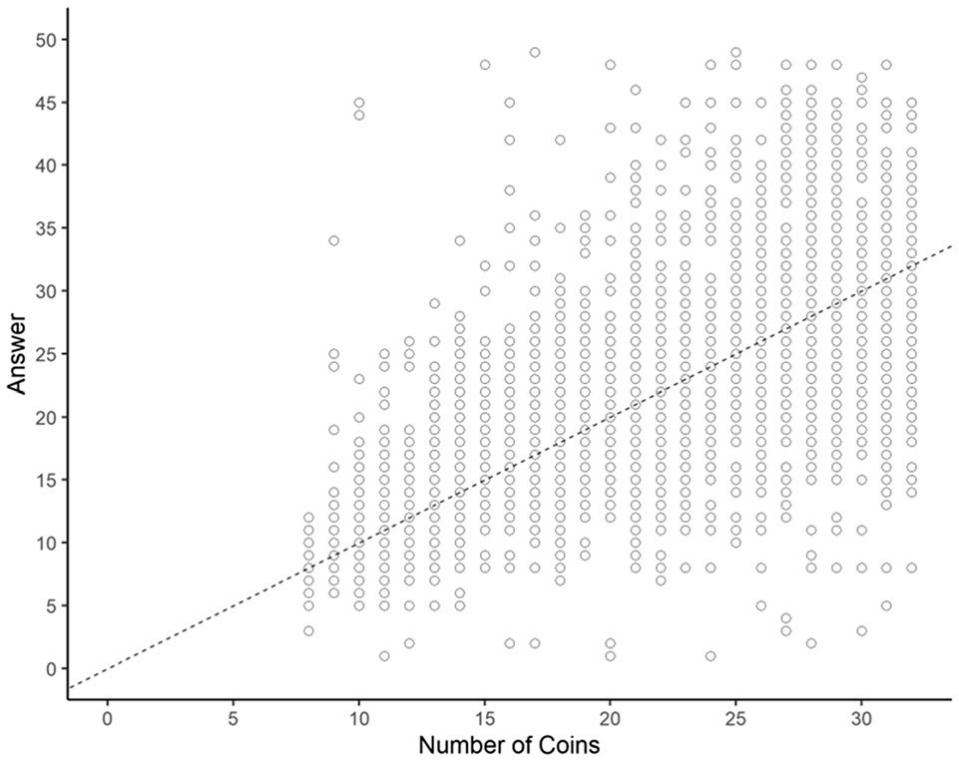
Performance on the coin count estimation task in Experiment 1 (scatter plot with the true number of coins on the x-axis and the estimated number of coins on the y-axis). The dotted line represents the case where the participant precisely estimated the true number of coins.

The distribution of participant responses is shown in Fig. 2. The distribution of data for individual participants is shown in Figs. S1–S6 of the Supplementary Information. The average coefficient of variation (CoV; the standard deviation of the number estimates divided by the true number of coins) was 0.179. This small value indicates that this task was not too difficult, given the value of 0.217 obtained in a previous study^16^ in which participants estimated the number of dots.

#### Single linear regression analysis

**Figure 3:**
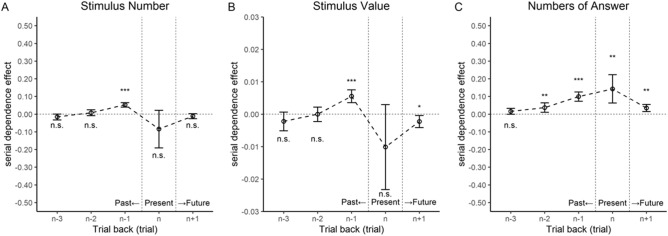
Serial dependence effects in Experiment 1. The y-axes represent the mean error in the participant responses. Positive errors indicate attractive serial dependence. (**A**) Serial dependence effects due to the number of coins presented in previous trials. (**B**) Serial dependence effects due to the monetary value of coins presented in previous trials. (**C**) Serial dependence effects due to the response (number) of the participant in previous trials. The results on the present trial are shown to identify errors due to the stimulus number, monetary value, and responses in previous trials. The results on the n + 1 trial are shown to confirm the autocorrelation of responses. Error bars indicate 95% confidence intervals. Significance levels in the figures are FDR-adjusted* p* values; n.s. = not significant, *p < 0.05, **p < 0.01, ***p < 0.001.

Figure 3A shows the serial dependence effects due to the number of coins on the preceding and following trials. Positive errors indicate overestimation of the number of coins on the current trial, while negative errors indicate underestimation of the number of coins on the current trial. The results of the single and multiple regression analyses are presented in Table 1. Significant positive average effects indicate attractive serial dependence, while significant negative average effects imply repulsive serial dependence. When preceding trials (from n − 3 to n − 1) were investigated, only the effect of the immediately preceding trial (n − 1) was significant. We also evaluated the effects of serial dependence on the current trial (n) and the immediately following trial (n + 1) as control conditions. No significant effects were observed on the n and n + 1 trials (Table 1). These results indicate that the number of stimuli in the immediately preceding trial produced attractive serial dependence.

**Table 1:** Summary of single and multiple regression analyses for Experiment 1. NoC indicates the number of coins, VoC indicates the monetary value of coins, and Ans indicates the participant’s response.

**Single linear regression analysis**									
**Factor**	**Average effect**	**(95% CI)**	**N**	**t value**	**d**	**p** **value**			R^2^
n − 1 NoC	0.053	(0.013)	22	8.51	1.77	0.000	***		0.008
n − 2 NoC	0.008	(0.017)	22	1.02	0.21	0.342	–		0.001
n − 3 NoC	− 0.016	(0.017)	22	− 1.95	− 0.41	0.108	–		0.000
n NoC	− 0.084	(0.106)	22	− 1.64	− 0.34	0.142	–		0.153
n + 1 NoC	− 0.011	(0.014)	22	− 1.66	− 0.35	0.142	–		0.000
n − 1 VoC	0.006	(0.002)	22	5.76	1.20	0.000	***		0.005
n − 2 VoC	0.000	(0.002)	22	− 0.04	− 0.01	0.969	–		− 0.001
n − 3 VoC	− 0.002	(0.003)	22	− 1.60	− 0.33	0.142	–		0.001
n VoC	− 0.010	(0.013)	22	− 1.61	− 0.33	0.142	–		0.102
n + 1 VoC	− 0.002	(0.002)	22	− 2.55	− 0.53	0.039	*		− 0.002
n − 1 Ans	0.100	(0.026)	22	7.97	1.66	0.000	***		0.035
n − 2 Ans	0.037	(0.027)	22	2.89	0.60	0.021	*		0.007
n − 3 Ans	0.017	(0.017)	22	2.02	0.42	0.104	–		0.002
n Ans	0.143	(0.08)	22	3.70	0.77	0.005	**		0.168
n + 1 Ans	0.035	(0.021)	22	3.52	0.73	0.006	**		0.005

**Multiple linear regression analysis**									
**Factor**	**Average effect**	**(95% CI)**	**N**	**t value**	**d**	**p** **value**		**VIF**	**adj.R2**
n − 1 NoC	− 0.089	(0.043)	22	− 4.29	− 0.90	0.001	***	7.28	0.203
n − 1 VoC	− 0.003	(0.003)	22	− 1.82	− 0.38	0.082	–	3.07	
n − 1 Ans	0.179	(0.049)	22	7.55	1.57	0.000	***	5.25	
n − 2 Ans	0.017	(0.016)	22	2.13	0.45	0.053	–	1.07	
n Ans	0.119	(0.079)	22	3.14	0.66	0.009	**	1.05	
n + 1 Ans	0.017	(0.014)	22	2.46	0.51	0.033	*	1.04	

In the stimulus set used in Experiment 1, even though the number of coins was held constant, the types of coins and the amounts differ. Therefore, we examined the effect of the monetary value of coins on the error. In Experiment 1, participants estimated the number of coins. Figure 3B shows the serial dependence effects due to the monetary value of coins on the preceding and following trials; only the effect of the immediately preceding trial (n − 1) was significant (Table 1). This result indicates that the monetary value of coins in the immediately preceding trial produced an attractive serial dependence effect on the current estimate of the number of coins. The effect of the n + 1 trial was also significant (Table 1); the error was significantly less than zero, indicating that the total monetary value on the next trial produced a repulsive serial dependence effect. The total monetary value of coins presented in each trial varied randomly; therefore, the interpretation of this effect is challenging. We consider the repulsive serial dependence effect to be due to chance and do not discuss it further.

We also examined the effect of participant responses in previous trials on errors in the current trial. Figure 3C shows the serial dependence effects due to participant responses in previous trials. The effects of responses from trials ranging from n − 1 to n + 1 were all significant (Table 1), indicating that participant responses from the n − 1, n − 2 and n − 3 trials produced attractive serial dependence. The effect of these past responses on the current trial confirmed the increase in estimation error in the positive direction with larger responses. The effect of responses on n + 1 trials also had a significant effect on the current trial: the larger the response in the next trial, the larger the estimation error in the current trial. Given the temporal order of events, however, this finding likely reflects the following effects: larger responses on the current trial result in larger subsequent (n + 1) responses and, simultaneously, larger estimation error on the current trial.

The analyses thus far confirm the existence of attractive serial dependence in the task involving estimation of the number of coins. However, comparison of (A) and (C) in Fig. 3 suggests that the previous responses had a stronger effect than the number of coins in previous trials. This pattern implies that serial dependence does not emerge in stimulus perception but rather in the process of generating a response based on stimulus perception.

To compare the effects of the number of coins and participant responses in previous trials more clearly, multiple regression analyses were conducted.

#### Multiple linear regression analysis

We conducted a multiple regression analysis, including variables that were significant in the single regression analysis. However, as noted above, the monetary value of coins in the n + 1 trial was not included in the analysis. Thus, the following multiple regression equation was used:

$$Error = intercept + \left[ {n - 1~NoC{:}x1} \right]\, +\, \left[ {n - 1~VoC{:}x2} \right] \,+\, \left[ {n - 1~Ans{:}x3} \right]\, + \,\left[ {n - 2~Ans{:}x4} \right] \,+\, \left[ {n~Ans{:}x5} \right] \,+ \,\left[ {n + 1~Ans{:}x6} \right]$$

where NoC indicates the number of coins, VoC indicates the monetary value of coins, Ans indicates participant responses, and the trial (preceding or following) is indicated by n ± the number of intervening trials from the current trial. For each participant, regression coefficients were calculated using this equation. The equation obtained by averaging the regression coefficients for all participants is as follows.

$$Error = ~ - 4.66 - 0.089*x1 - 0.003*x2 + 0.179*x3 + 0.017*x4 + 0.011*x5 + 0.119*x6 + 0.017*x7$$

Each regression coefficient was evaluated using a one-sample *t* test that compared its value against the null hypothesis that the mean of the regression coefficients did not differ from 0. All but the monetary value of coins in the n − 1 trial (n − 1 VoC) and the responses in the n − 2 trial (n − 2 Ans) were significant, and the null hypothesis was rejected. As shown in Table 1, the n − 1 trial responses (n − 1 Ans) had the strongest serial dependence effect. Unlike the results of the single regression analysis, the coefficient of the number of coins in the previous trial (n − 1 NoC) was negative. In other words, when the effects of the number of coins and responses were analyzed simultaneously, the number of coins in the preceding trial produced a repulsive serial dependence effect.

The change in the sign of the n − 1 NoC coefficient in the multiple regression analysis suggests that the attractive serial dependence observed in the single regression analysis might have been caused by the correlation between the number of coins and responses in the n − 1 trial.

The effect of the monetary value of coins on the response in the current trial in the multiple regression analysis was not significant. The significant effect of the monetary value of coins in the single regression analysis may have also been caused by the correlation with the response in the n − 1 trial.

The total number of coins, the total value of coins, and the participants' responses in the same trial inevitably correlated with each other. Therefore, multicollinearity might have affected the results of the multiple regression analysis in Experiment 1. We therefore calculated the variance inflation factor (VIF)^29^ for each participant and presented the mean values in Table 1. For all explanatory variables, the VIF was below 10, which is considered a critical value. We further conducted a ridge regression^30^ and compared the results with Table 1; as shown in Table S1 in the Supplementary Information, the results of the ridge regression were similar to those of Table 1. No change in the sign of the explanatory variables was observed. These results suggest that multicollinearity did not seriously affect the results of the multiple regression analysis. See Supplemental information for details on the ridge analysis. The correlation matrix between all explanatory and objective variables entered into the multiple regression analysis is shown in Fig. S13.

### Experiment 2

Next, participants were asked to estimate the monetary value of coins presented on the screen. As in Experiment 1, the stimulus duration was 500 ms, and the number of coins ranged from 8 to 32. This presentation made it impossible for participants to accurately count the number of coins of each type and calculate the total monetary value. Participants thus answered by estimating the monetary value of coins.

#### Task performance

**Figure 4:**
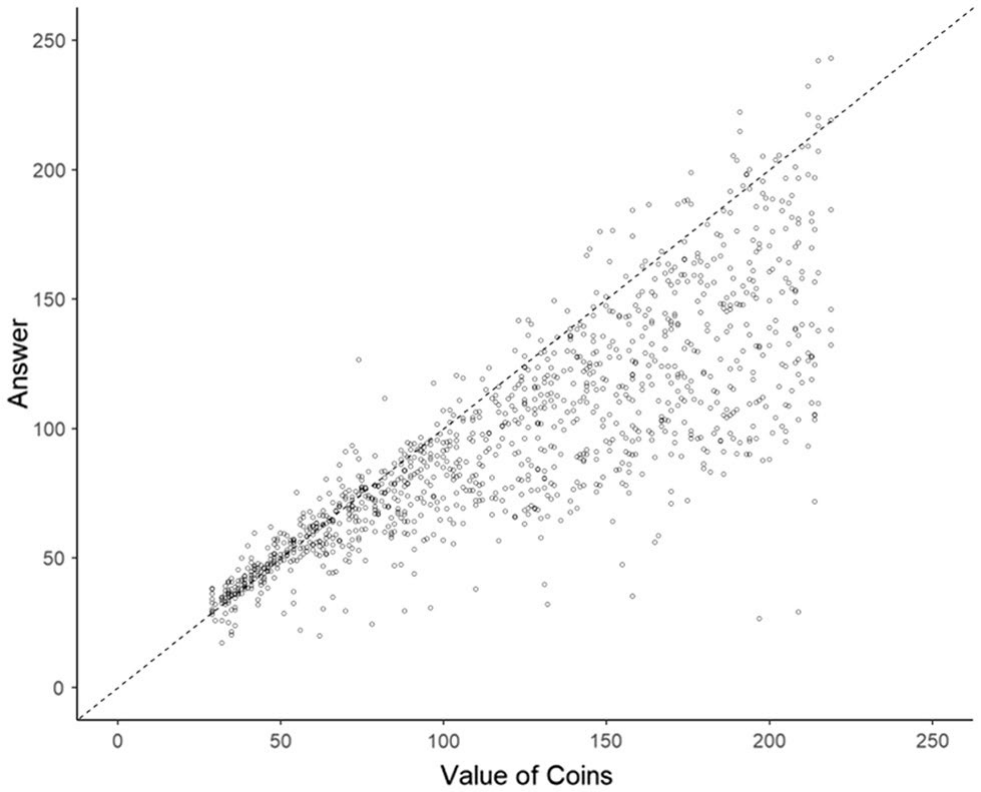
Performance on the monetary value estimation task in Experiment 2 (scatter plot with the true monetary value of the coins on the x-axis and the participant responses on the y-axis). The dotted line represents the case where the participant precisely estimated the true monetary value of the coins.

As shown in Fig. 4, the size of participant responses increased as the monetary value of coins increased. The distribution of data for individual participants is shown in Figs. S7–S12 of the Supplementary Information. The average CoV, the standard deviation of total value estimates divided by the true total value of the coins, was 0.208. This value is larger than that in Experiment 1; however, it is smaller than the values in a previous study^16^ where participants estimated the number of dots. Therefore, we did not consider this task too difficult.

#### Single linear regression analysis

**Figure 5:**
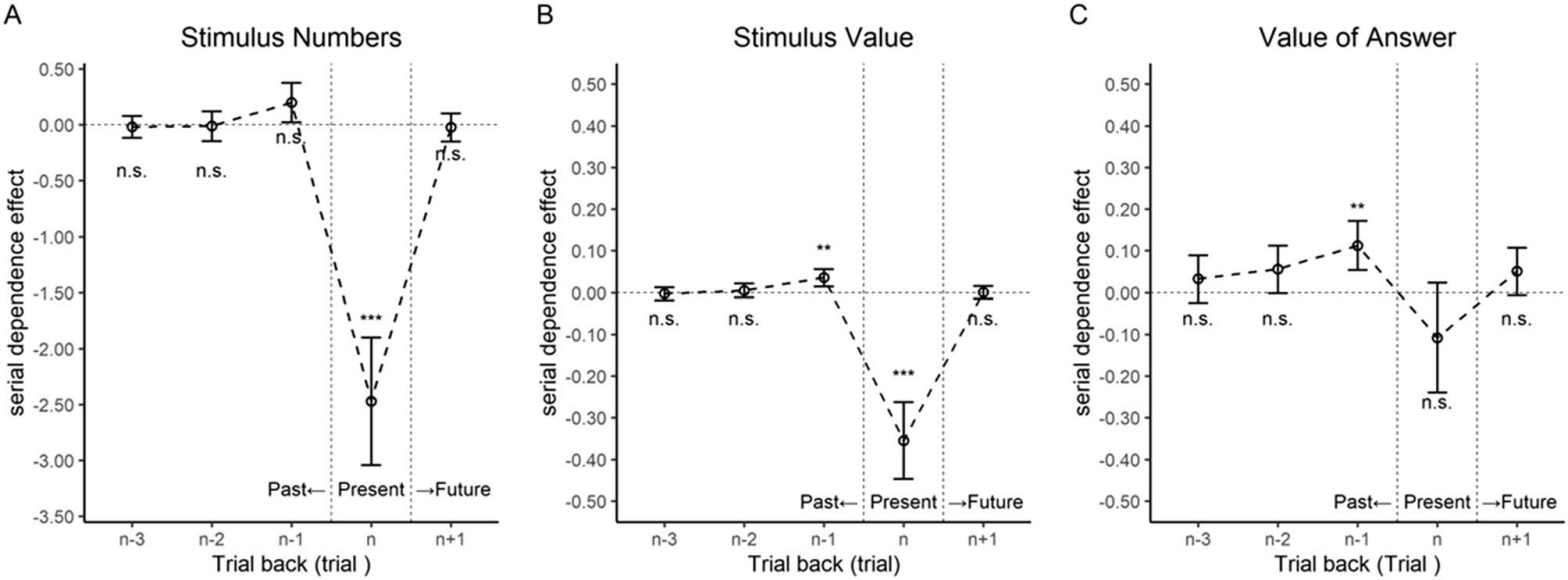
Serial dependence effects in Experiment 2. The y-axes represent the mean error in the value provided by the participants. Positive errors indicate attractive serial dependence. (**A**) Serial dependence effects due to the number of coins presented in previous trials. (**B**) Serial dependence effects provided by the monetary value of coins presented in previous trials. (**C**) Serial dependence effects due to the participant response (value) in previous trials. The results on the present trial are shown to identify errors due to the stimulus number, monetary value, and participant responses in the previous trial. The results on the n + 1 trial are shown to confirm the autocorrelation of responses. Error bars indicate 95% confidence intervals. Significance levels in the figures are FDR-adjusted *p* values; n.s. = not significant, *p < 0.05, **p < 0.01, ***p < 0.001.

As in Experiment 1, we conducted an analysis in which the number of coins was an explanatory variable because the number of coins presented could affect the estimation of the total monetary value. Figure 5A shows the serial dependence effects due to the number of coins on the preceding and following trials. A significant effect of the number of coins was observed only on the current trial (Table 2). This result suggests that more coins are displayed, the larger the underestimation of the monetary value of these coins. This finding is likely because the more coins are displayed in the current trial, the more difficult it is to estimate the total monetary value, resulting in increased estimation error.

**Table 2:** Summary of the single and multiple regression analyses for Experiment 2. NoC indicates the number of coins, VoC indicates the monetary value of coins, and Ans indicates the participant’s response.

**Single linear regression analysis**									
**Factor**	**Average effect**	**(95% CI)**	**N**	**t value**	**d**	**p** **value**		**R**^2^	
n − 1 NoC	0.199	(0.177)	22	2.33	0.49	0.088	–	0.005	
n − 2 NoC	− 0.012	(0.134)	22	− 0.18	− 0.04	0.917	–	0.000	
n − 3 NoC	− 0.018	(0.097)	22	− 0.38	− 0.08	0.833	–	− 0.001	
n NoC	− 2.471	(0.571)	22	− 8.98	− 1.87	0.000	***	0.346	
n + 1 NoC	− 0.023	(0.126)	22	− 0.38	− 0.08	0.833	–	− 0.001	
n − 1 VoC	0.036	(0.021)	22	3.57	0.74	0.006	**	0.005	
n − 2 VoC	0.005	(0.016)	22	0.65	0.14	0.781	–	0.000	
n − 3 VoC	− 0.003	(0.016)	22	− 0.36	− 0.08	0.833	–	− 0.001	
n VoC	− 0.355	(0.092)	22	− 7.99	− 1.67	0.000	***	0.414	
n + 1 VoC	0.001	(0.015)	22	0.08	0.02	0.933	–	− 0.001	
n − 1 Ans	0.113	(0.059)	22	3.98	0.83	0.003	**	0.030	
n − 2 Ans	0.056	(0.057)	22	2.04	0.43	0.133	–	0.016	
n − 3 Ans	0.032	(0.057)	22	1.17	0.24	0.427	–	0.013	
n Ans	− 0.108	(0.132)	22	− 1.70	− 0.35	0.194	–	0.106	
n + 1 Ans	0.051	(0.057)	22	1.86	0.39	0.164	–	0.016	

**Multiple linear regression analysis**									
**Factor**	**Average effect**	**(95% CI)**	**N**	**t value**	**d**	**p** **value**		**VIF**	**adj.R2**
n − 1 VoC	− 0.069	(0.034)	22	− 4.20	− 0.88	0.000	***	4.21	0.464
n − 1 Ans	0.167	(0.067)	22	5.18	1.08	0.000	***	4.21	
n VoC	− 0.260	(0.104)	22	− 5.17	− 1.08	0.000	***	3.52	
n NoC	− 0.812	(0.268)	22	− 6.29	− 1.31	0.000	***	3.53	

Figure 5B shows the serial dependence effects due to the monetary value of coins on the preceding and following trials; only the effect of the immediately preceding trial (n − 1) was significant (Table 2). This result indicates that the monetary value of coins in the immediately preceding trial produced an attractive serial dependence effect on the estimation of the monetary value of coins, suggesting that serial dependence originates in higher-order processing in which calculation takes place. The effect of n VoC was also significant, confirming that estimation error increased as the monetary value of coins in the present trial increased. This result is consistent with the trend observed in Fig. 2, where underestimation increases as the monetary value of coins increases. No other significant effects were observed.

We also examined the effect of participant responses in previous trials on errors in the current trial. Figure 5C shows the serial dependence due to responses in previous trials. A significant effect was observed only in the n − 1 trial (Table 2), indicating that the responses in n − 1 trials produced an attractive serial dependence effect on the current response.

#### Multiple linear regression analysis

We conducted a multiple regression analysis, including all significant variables from the single regression analysis. The following multiple regression equation was used:

$$Error=intercept+\left[n-1 VoC{:}x1\right]+\left[n-1 Ans{:}x2\right]+\left[n VoC{:}x3\right]+\left[n NoC{:}x4\right]$$

where VoC indicates the monetary value of coins, Ans indicates the participant response, NoC indicates the number of coins, and the trial (preceding or following) is indicated by n ± the number of intervening trials from the current trial. For each participant, regression coefficients were calculated using this equation. The equation obtained by averaging the regression coefficients for all participants is as follows.

$$Error = ~18.59 - 0.069*x1 + 0.167*x2 - 0.260*x3 - 0.812*x4$$

Each regression coefficient was evaluated using a one-sample *t* test to compare its value against the null hypothesis that the mean of the regression coefficients did not differ from 0. All regression coefficients were significant, and the null hypothesis was rejected (Table 2).

The effects of n − 1 Ans and the effects of the number of coins and total monetary value of coins on the current trial had the same sign of coefficients as in the single regression analyses. In contrast, the sign of the effect of n − 1 VoC was reversed compared to the single regression analysis, indicating repulsive serial dependence. This result is similar to the effect of n − 1 NoC in Experiment 1, suggesting that the attractive serial dependence effect of n − 1 VoC observed in the single regression analysis may have been caused by the correlation between the response and the monetary value of coins in the n − 1 trial.

As in Experiment 1, the number of coins, the total value of coins, and the response on the same trial were correlated with each other in Experiment 2 (Table S14). Therefore, as in Experiment 1, VIF was calculated, and ridge analysis was conducted; as shown in Table 2, VIF was below 10 for all explanatory variables. The result of the ridge regression (Table S2) was similar to that in Table 2. No change in the sign of the explanatory variables was observed. These results suggest that multicollinearity did not seriously affect the multiple regression analysis of Experiment 2. See Supplemental information for details on the ridge analysis. The correlation matrix between all explanatory and objective variables entered into the multiple regression analysis is shown in Fig. S14.

## Discussion

Attractive serial dependence has been reported to occur with a variety of stimuli, including line tilt^2^, motion^8^, faces^9^, number perception^1,6^, and sense of agency^10^. These phenomena are similar in that they make the experience of current stimuli similar to that of past experiences. However, the representation that causes serial dependence is thought to differ according to stimulus. In the task involving adjustment to the tilt of the Gabor patch^2,31^, the information held in the perceptual system is presumed to influence the tilt judgment of the current stimulus. In the task of involving guesses of the number of dots, the number is thought to be estimated according to the density and number of stimuli as well as the range they occupy in the visual field by a system specific to number perception^24^; the information held about previous responses is also presumed to influence current number judgments. Representation of numbers is considered more abstract than representation of the tilt of a line. The persistence of serial dependence across stimuli despite differences in processing requirements suggests that this phenomenon reflects a universal property of systems that estimate features of the external world. However, whether this universal property originates from the initial perceptual processing, higher-order perceptual processing, or the process of generating responses remains unclear.

One of the objectives of this study was to verify whether serial dependence occurs in tasks that require higher-order processing, i.e., estimating the total monetary value of coins. Using coins as stimuli allowed us to confirm the presence of serial dependence effects in the number of coins and the monetary value of coins with the same stimuli. The task of estimating the monetary value of the coins required estimating the number of coins of each type, multiplying the estimated number by the denomination of each coin, and summing the results; this task thus required higher-order processing than the simple estimation of the total number of coins.

We found serial dependence due to the number of coins in Experiment 1 and serial dependence due to the monetary value of the coins in Experiment 2; both of these effects were attractive. The presence of a serial dependence effect in monetary value, which is determined not only by the number of stimuli but also by culturally defined attributes of the stimuli, suggests that serial dependence occurs at the level of higher-order processing (i.e., number representations required for calculation). This result is consistent with reports that attractive serial dependence also occurs between stimuli represented in different formats, such as the number of flashes and the number of dots, and suggests that serial dependence occurs at the level of abstract numbers^14,17^.

In the studies of number perception, it has been known that for small numbers, participants can respond with high accuracy by subitization. However, as the number increases, the error increases linearly. Attractive serial dependence in number perception means that estimates of the number of objects in the current stimulus are induced to the past stimuli. When the number of objects in the environment changes continuously, this effect allows the organism to obtain the correct value at a low cost. This is a similar view to that of Pascucci et al.'s decisional templates for tilt perception.

As mentioned in the Introduction, it is known that the proximity in the feature space of the preceding and following stimuli and spatial attention to preceding stimuli affect serial dependence. Neither of these factors were controlled in this study. Because the stimuli used in Experiments 1 and 2 were randomly generated, we think it unlikely that they had a bias in any particular direction with respect to similarity between the preceding and following stimuli or attention to the stimulus. However, the possibility that some bias may have occurred cannot be ruled out, and it will be necessary to control for these factors in future studies.

Because participants in this study were required to respond to all stimuli, the time interval between the preceding and following stimuli varied depending on the reaction time of the task. Although reaction time was not measured in this study, it is likely that as the number and value of coins increased, the difficulty of the task increased, and therefore, the reaction time also increased. It is thus possible that the effect of the number and value of coins was confounded by the effect of the time interval between the preceding and following stimuli. This point also needs to be examined in future studies.

It should be noted that there was a positive correlation (*r* = 0.84) between the number of coins and the monetary value of coins in the stimuli of Experiment 2, although we designed the stimuli to be as varied as possible in terms of value represented the same number of coins (see “Methods”). This design ensured a range of variation in the monetary value independent of the number of coins (Fig. 1) and allowed us to independently analyze the effects of the number and monetary value of coins on estimation error through multiple regression analysis. The correlation between the number of coins and the monetary value of coins was inevitable given our aim to provide variation in the number and monetary value of coins. As noted in the results of Experiments 1 and 2, the correlation could have caused the multicollinearity problem. Since the multiple regression analyses of the results of both experiments showed that the values of VIF were less than 10, which is considered critical, and the results of ridge analyses showed the equivalent tendencies, it is likely that multicollinearity did not substantially affect the results of multiple regression analyses in this study. However, to exclude the possibility of multicollinearity, it will be necessary to conduct experiments with stimuli that eliminate correlations between the number and value of coins.

Another objective of this study was to examine the effect of past responses on serial dependence. We used multiple regression analysis to simultaneously analyze the effects of previous stimuli and previous responses. We found that in both Experiments 1 and 2, the immediate preceding response exerted an attractive effect on the response in the current trial, while the immediate preceding stimuli exerted a repulsive effect. This finding suggests that the attractive serial dependence effect of previous stimuli on the current trial observed in the single regression analysis was actually due to the correlation between stimuli and responses in previous trials and that serial dependence is influenced by the strength of the participant's internally generated perception or response rather than by the attributes of the external stimulus.

In a study on serial dependence in tilt perception, participants' past responses produced an attractive serial dependence effect, and past stimuli produced a repulsive serial dependence effect, which is equivalent to adaptation to the stimuli^3^. Adaptation in number perception has been reported in a study that measured the point of subjective equality (PSE) of the number of dots^32^. Their results are consistent with our own. Pascucci et al. (2019) explained the emergence of serial dependence by postulating the existence of two processes: adaptation, which is assumed to occur over a short time period during feature detection in lower-order processing, and perceptual decision-making, which occurs after the accumulation of sensory evidence^13^. They argued that the existence of two processes with different time scales, namely, a process that efficiently creates internal representations of the percepts and one that generates responses to a continuously changing environment, enable adaptive perception and decision. Moreover, Fritsche et al. (2020) showed that previous experimental results can be reproduced by a Bayesian model based on this framework^3,12^.

In studies of serial dependence in numerosity perception, the relationship between the past response and the present response has not been fully explored. In Experiment 1, participants were asked to estimate the number of coins, and multiple regression analysis revealed both the attractive effect of the past response and the repulsive effect of the past stimulus. In future studies of numerosity perception, it is necessary to examine whether similar effects are observed in experiments where participants estimate the number of dots.

The present study showed that attractive serial dependence due to past responses also occurs in the estimation of the number or total monetary value of coins. This finding suggests that processes that utilize previous perceptual decisions in the next decision occur in various types of perception. Such decision inertia has been reported in cases when perceptual evidence is ambiguous^26^. Further studies are needed to determine whether the effects of past responses are caused by ambiguity in perceptual evidence or whether they also occur when perceptual ambiguity is low or absent.

## Conclusion

We examined the effects of serial dependence in a task involving estimates of the total number of coins (Experiment 1) and the monetary value of coins (Experiment 2) displayed. In both tasks, the attractive effects of the number of coins or the monetary value of coins in the previous trial were confirmed in single regression analyses. The detection of serial dependence in the monetary value estimation task suggests that serial dependence originates in higher-order cognitive processing that performs calculation. We also examined the effect of past responses on serial dependence by conducting multiple regression analyses to simultaneously analyze the effects of previous stimuli and previous responses. In both number estimation and monetary value estimation, the immediately preceding responses exerted an attractive effect on responses in the current trial, whereas the immediately preceding stimuli exerted a repulsive effect. This pattern suggests that the attractive serial dependence due to previous stimuli observed in the single regression analyses was actually due to the correlation between stimuli and responses in the previous trials and that the effect of past stimuli per se is an adaptation that increases sensitivity to current stimuli. The emergence of attractive serial dependence due to past responses is consistent with the view that serial dependence originates in higher-order cognitive processing.

## Methods

We investigated serial dependence in two experiments using coins as stimuli. In Experiment 1, participants were asked to estimate the total number of coins presented on the screen. In Experiment 2, participants were asked to estimate the monetary value of coins presented on the screen. The same participants participated in both Experiments 1 and 2 successively on the same day. The order of the experiments was counterbalanced across participants.

### Experiment 1

A total of 24 subjects participated in the study (7 males, mean age = 20.21, SD = 3.78 years). Two of the participants were excluded from the analysis because they demonstrated insufficient understanding of the experimental task based on their data. Subjects were compensated with course credits for their participation. All participants had normal or corrected-to-normal vision and provided written informed consent prior to taking part in the study. All the experimental procedures were approved by the Ethics Committee of the Graduate School of Humanities and Sustainable System Science, Osaka Prefecture University, and was performed in accordance with the latest version of the Declaration of Helsinki. The number of participants was determined based on the number of participants in similar studies^14^.

#### Apparatus and stimulus

All experiments were performed using MATLAB (version 2021a, The MathWorks Inc., Natick, MA, USA) and Psychtoolbox-3^33–35^ running on a computer with a Intel Core 7-8700 CPU and a GeForce GTX 1080Ti 11 GB GDDR5X GPU with an Ubuntu 20.04LTS operating system. Participants were presented with stimuli at a viewing distance of approximately 50 cm from the 24-inch monitor (ASUS VG248QE; resolution: 1920 × 1080 pixels). The refresh rate of the monitor was set to 99.89 Hz, and a USB keyboard was used to acquire responses.

The screen background was gray, and the instructions were presented in white font. The fixation point was a black cross (+) (20 × 20 pixels). The stimuli consisted of images of a 1-yen coin (86 × 86 pixels), a 5-yen coin (98 × 98 pixels), and a 10-yen coin (104 × 104 pixels). The images of the coins were not photographs of actual coins but rather drawings that highlighted the characteristics of each type of coin. Coin images were presented at a size that maintained the same ratio as the actual coin sizes. The total number of coins on the screen had 25 possible amounts (ranging from 8 to 32 coins), and the number of coins of each type was randomly determined on each trial. The screen was divided into virtual cells in a 6 × 6 pattern, and each coin was placed in a randomly selected cell. The position of the coins in the cell was randomly jittered so that the entire array of coins differed from trial to trial. Each cell consisted of a 150 × 150 pixel square, and a coin was presented in the center of the grid if the jitter value was 0. The jitter (within ± 20 pixels) was randomly determined for each grid so that the coins did not overlap. An example of the coin display is shown in Fig. 1. The maximum and minimum monetary values were ¥249 and ¥10, respectively. There were 222 amounts.

#### Procedure

The experimental procedure was designed with reference to that of Fornaciai & Park (2020). In Experiment 1, participants were asked to estimate the total number of coins on the screen. The experiment began after participants were informed that the task was to estimate the total number of coins. On each trial, a fixation point was first presented in the center of the screen for 500 ms, followed by a target stimulus consisting of multiple coins for 500 ms. Then, the prompt "? >> " was presented. The participants were instructed to report the estimated number of coins by entering the number on a keyboard. No time limit was imposed on the response period. The numbers entered by the participants were shown on the screen, and they could correct their responses by pressing backspace. Once input was complete, participants were instructed to press the Enter key to confirm their response, and after a blank screen, the next trial was automatically initiated. The duration that the blank screen was displayed varied randomly in the range of 1350–1450 ms.

Participants were instructed that the possible range of the number of coins displayed was between 4 and 36 coins; this range was wider than the actual range. This instruction was added to induce uncertainty in participant responses in displays containing the minimum (8) and maximum (32) number of coins. Participants completed five blocks of 50 trials, for a total of 250 trials. For each participant, stimuli consisting of one of possible amounts of the number of coins from 8 to 32 (i.e., 25 possible amounts) were presented 10 times. Even when the number of coins was the same across trials, the ratio of the number of the three coin types (1-yen, 5-yen, and 10-yen) varied randomly. To exclude typing errors from the analysis, responses less than 0 or greater than 50 were excluded from the analysis.

Participants were allowed to take a 30-s break between blocks. Before starting the experimental trials, participants completed 25 practice trials. The procedure of the practice trials was identical to that of the experimental trial. Participants participated in Experiments 1 and 2 on the same day; the total duration of the two experiments was approximately 90 min.

### Experiment 2

#### Participants, apparatus, and stimuli

The participants, coin images, and apparatus were the same as in Experiment 1, except for the generation of the stimulus set. The stimulus set was generated by the following procedure. First, 100,000 sets of coins were randomly generated such that the total number of coins ranged from 8 to 32, the same as in Experiment 1. All sets consisted of the three types of coins, and duplication of identical sets was allowed. Next, the 100,000 sets were classified by their monetary values. To manipulate the number and monetary value of coins as independently as possible, 186 amounts of monetary values with five or more variations in the number of coins were selected to use as monetary values in Experiment 2. From these 186 amounts of monetary values, 50 amounts were selected for each participant. Stimuli were then created for each participant to ensure that for each of the 50 amounts of monetary values, each participant received 5 trials with randomly selected numbers of coins, for a total of 250 trials. Stimuli were selected such that no stimulus consisted of the same number of coins and the same monetary value. This method ensured variation in the number of coins for each monetary value. The maximum and minimum monetary values were ¥219 and ¥29, respectively.

#### Procedure

In Experiment 2, participants were asked to estimate the monetary value of the coins on the screen. The experiment began after participants were informed that the task was to estimate the monetary value of the displayed coins. To exclude typing errors from the analysis, responses less than 0 or greater than 500 were excluded from the analysis. The other procedures were the same as in Experiment 1.

## Supplementary Information

Supplementary Information.

## Data Availability

The datasets collected in this study are available from the corresponding author on reasonable request.
