# Supplementary material for: Serial dependence in estimates of the monetary value of coins
Source: Sci Rep. 2022 Nov 23;12:20212. doi: 10.1038/s41598-022-24236-z (PMC9684444; doi:10.1038/s41598-022-24236-z)
Supplement: Supplementary file 1 — Supplementary Information. [file 41598_2022_24236_MOESM1_ESM.pdf]

Supplementary Information

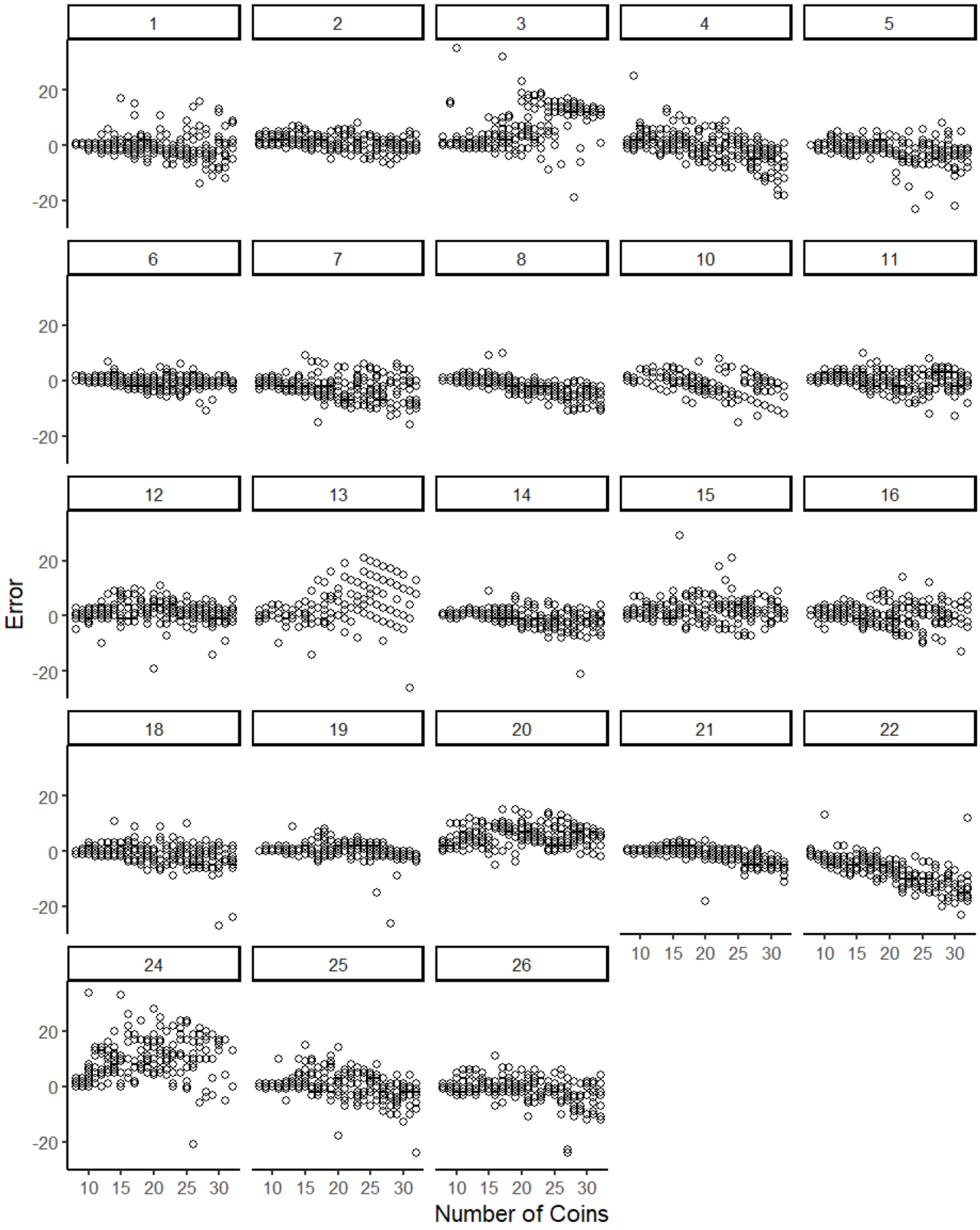

Figure S1. Distribution of errors as a function of the number of coins in the current trial in Experiment 1.

The circles in the graph indicate the error value for each participant on each trial. The numbers in the rectangles represent the participant number.

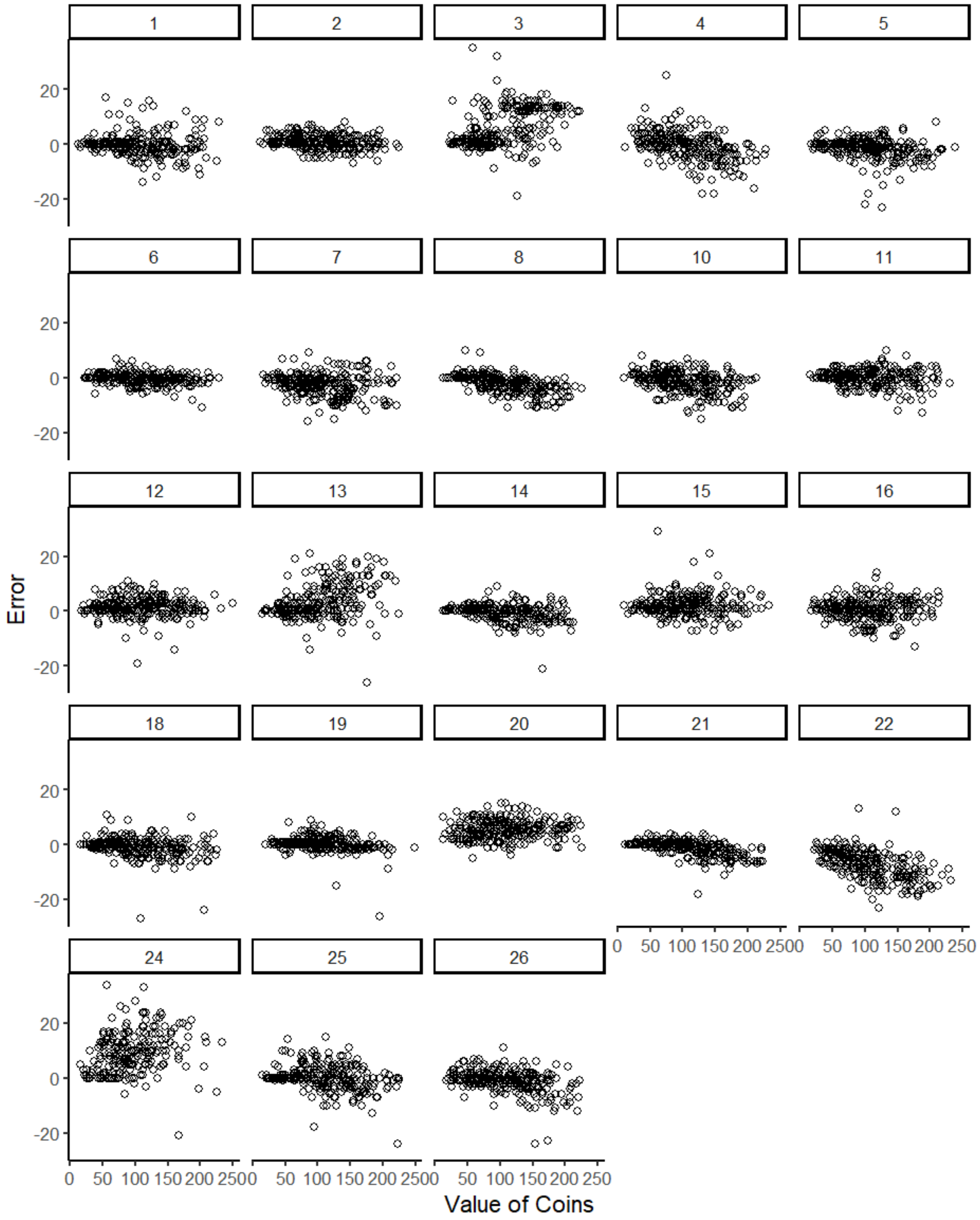

Figure S2. Distribution of errors as a function of coin value in the current trial in Experiment 1. The circles in the graph indicate the error value for each participant on each trial. The numbers in the rectangles represent the participant number.

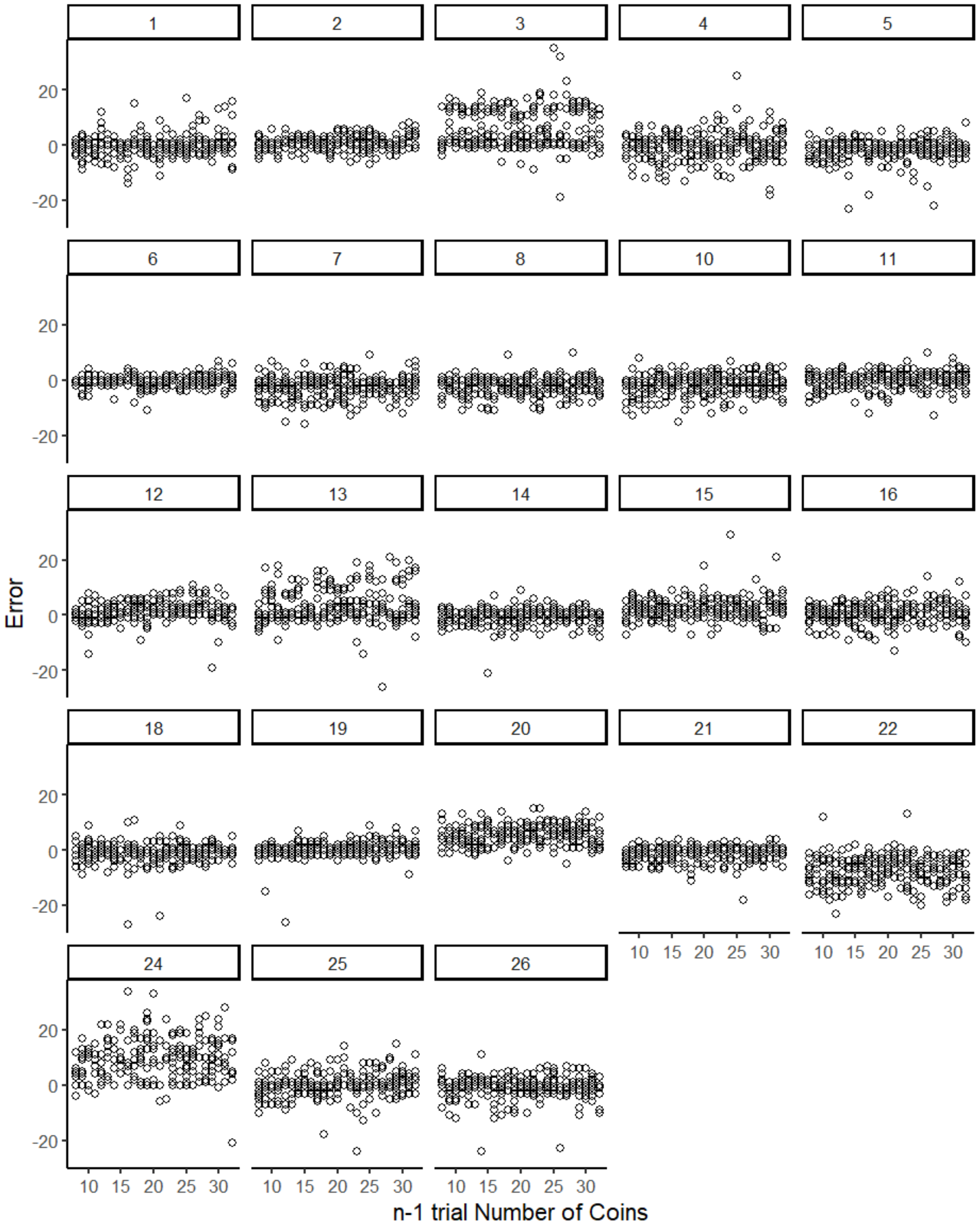

Figure S3. Distribution of errors as a function of the number of coins in trial n-1 in Experiment 1. The circles in the graph indicate the error value for each participant on each trial. The numbers in the rectangles represent the participant number.

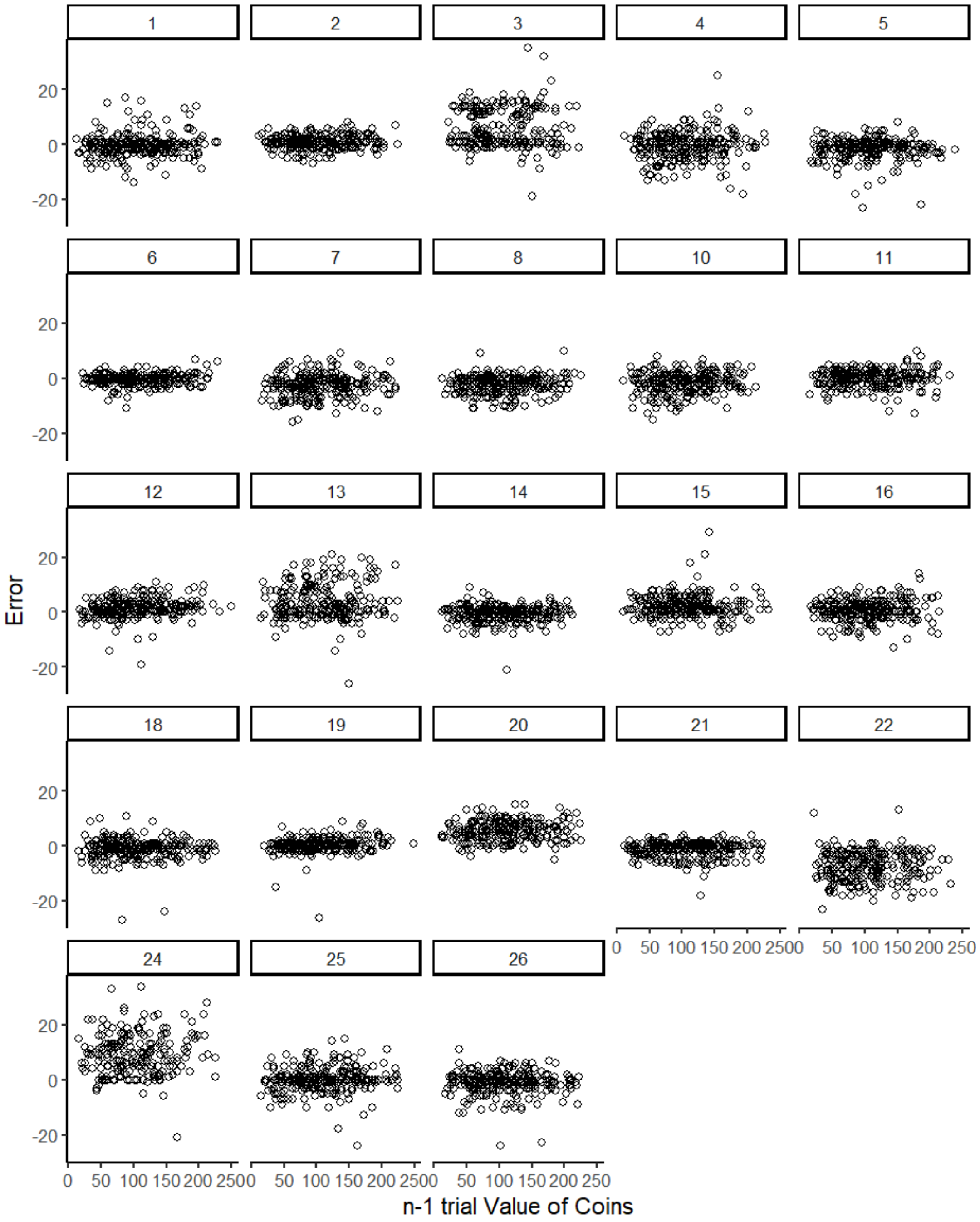

Figure S4. Distribution of errors as a function of coin value in trial n-1 in Experiment 1.
The circles in the graph indicate the error value for each participant on each trial. The numbers in the rectangles represent the participant number.

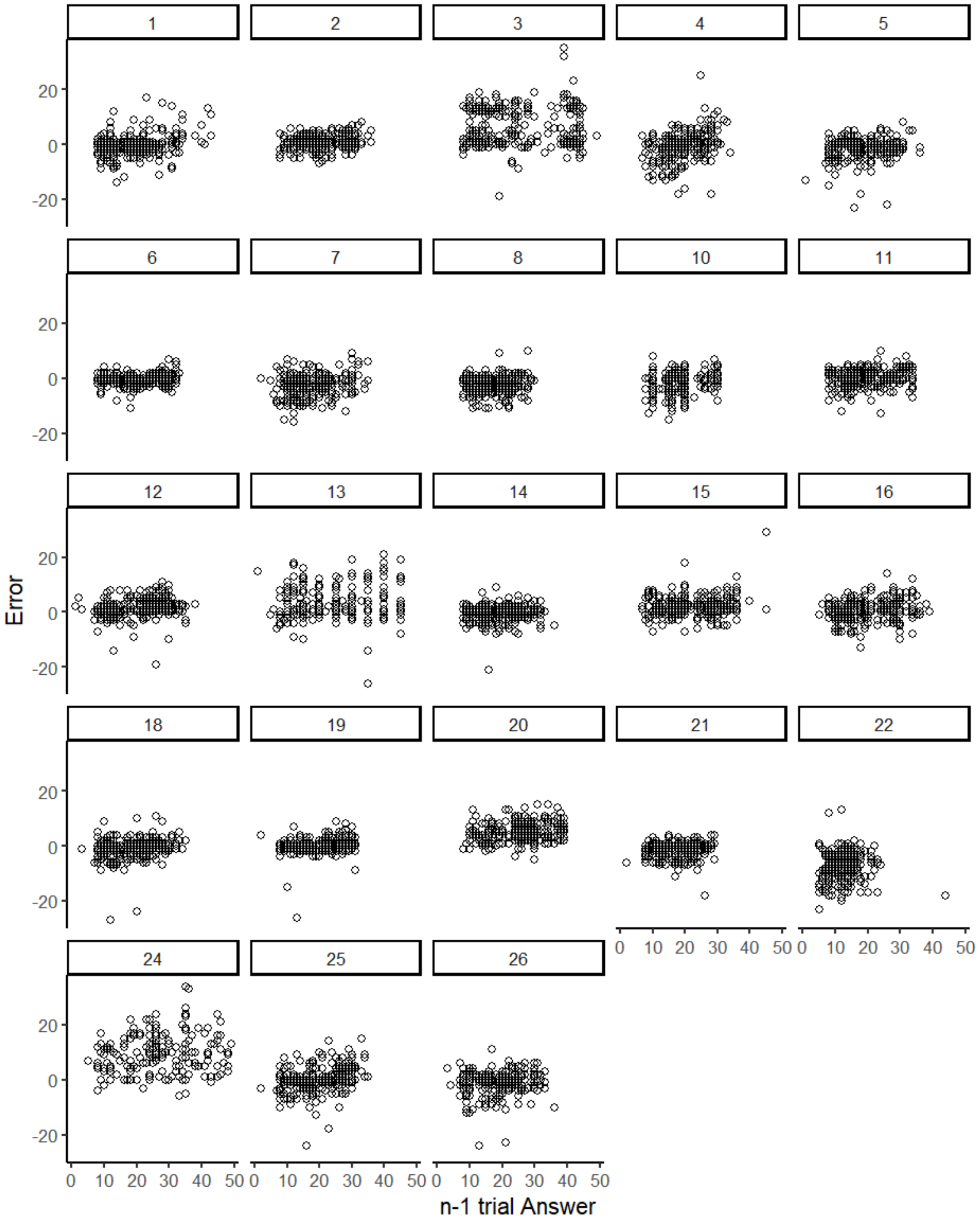

Figure S5. Distribution of errors as a function of the value of coins answer in trial n-1 in Experiment 1.

The circles in the graph indicate the error value for each participant on each trial. The numbers in the rectangles represent the participant number.

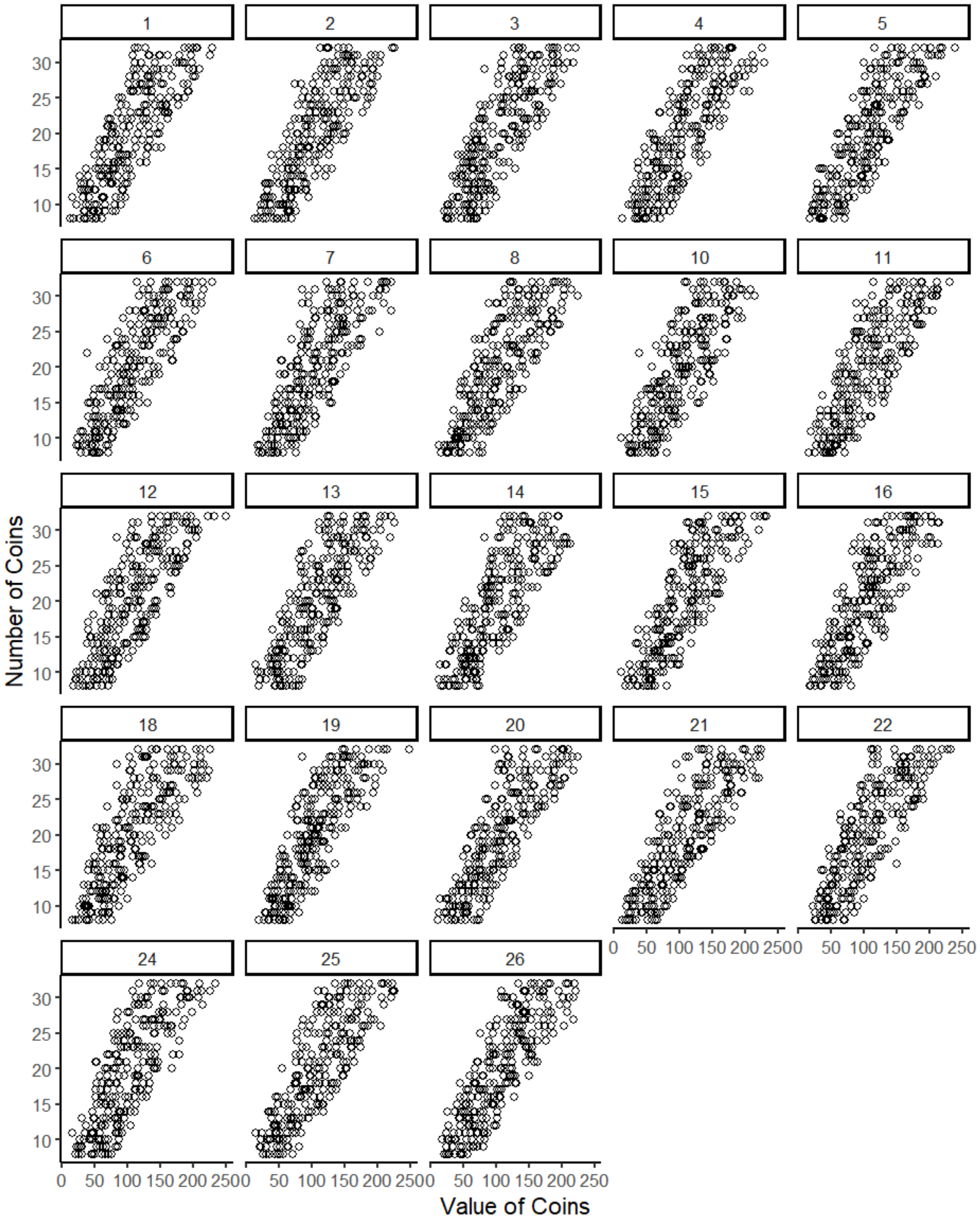

Figure S6. Distribution of the number of coins as a function of coin value in the current trial in Experiment 1.

The circles in the graph indicate the error value for each participant on each trial. The numbers in the rectangles represent the participant number.

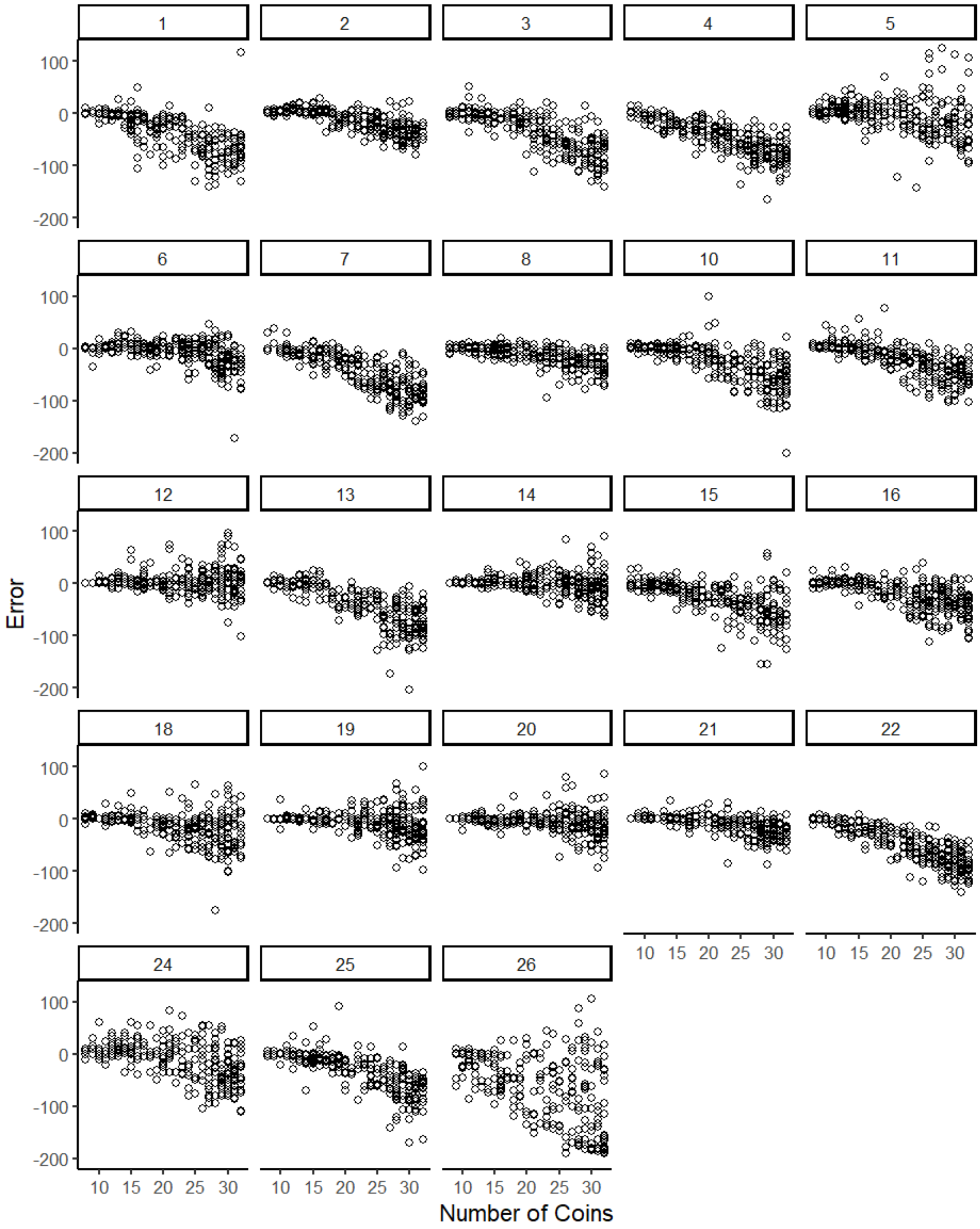

Figure S7. Distribution of errors as a function of the number of coins in the current trial in Experiment 2.

The circles in the graph indicate the error value for each participant on each trial. The numbers in the rectangles represent the participant number.

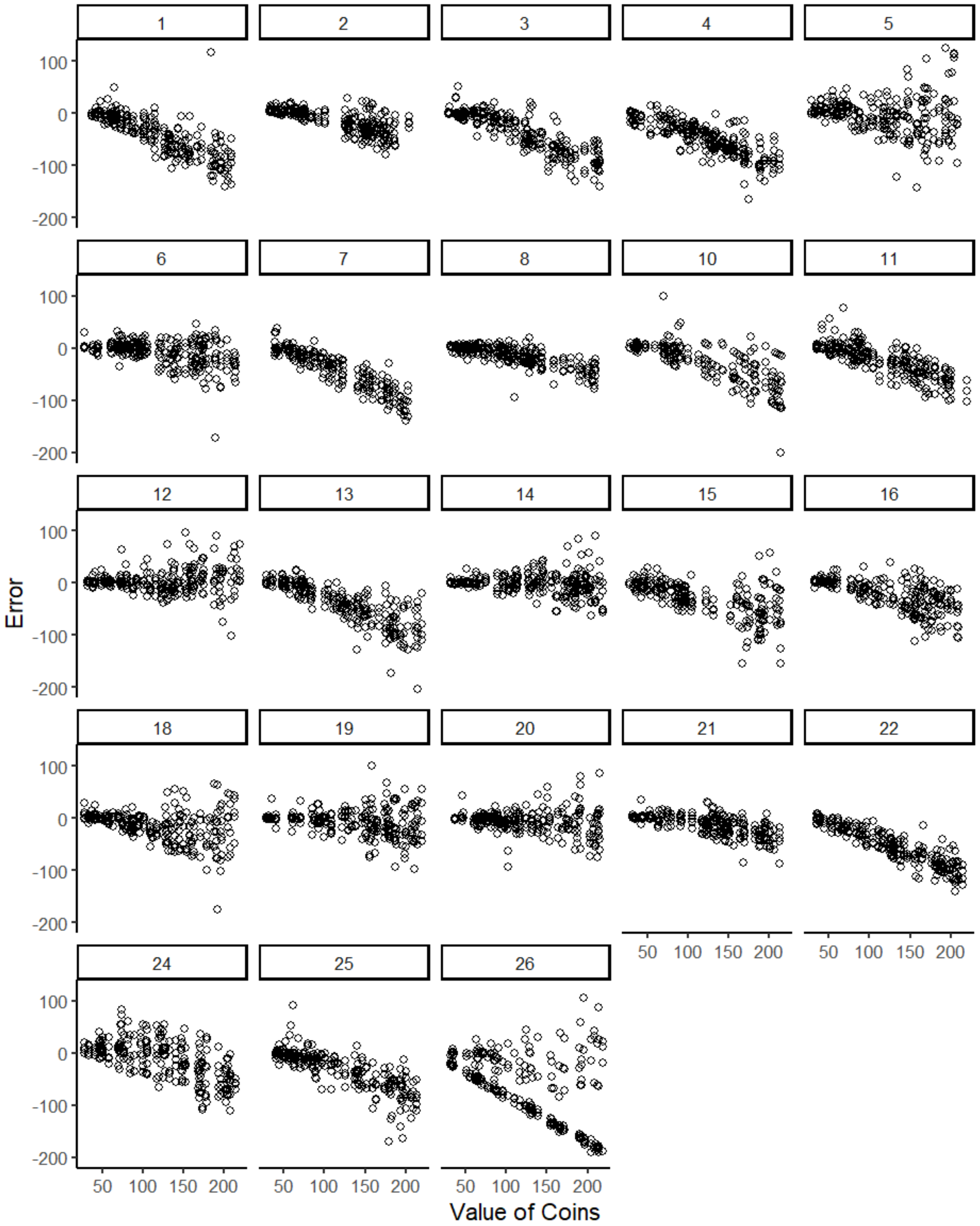

Figure S8. Distribution of errors as a function of coin value in the current trial in Experiment 2. The circles in the graph indicate the error value for each participant on each trial. The numbers in the rectangles represent the participant number.

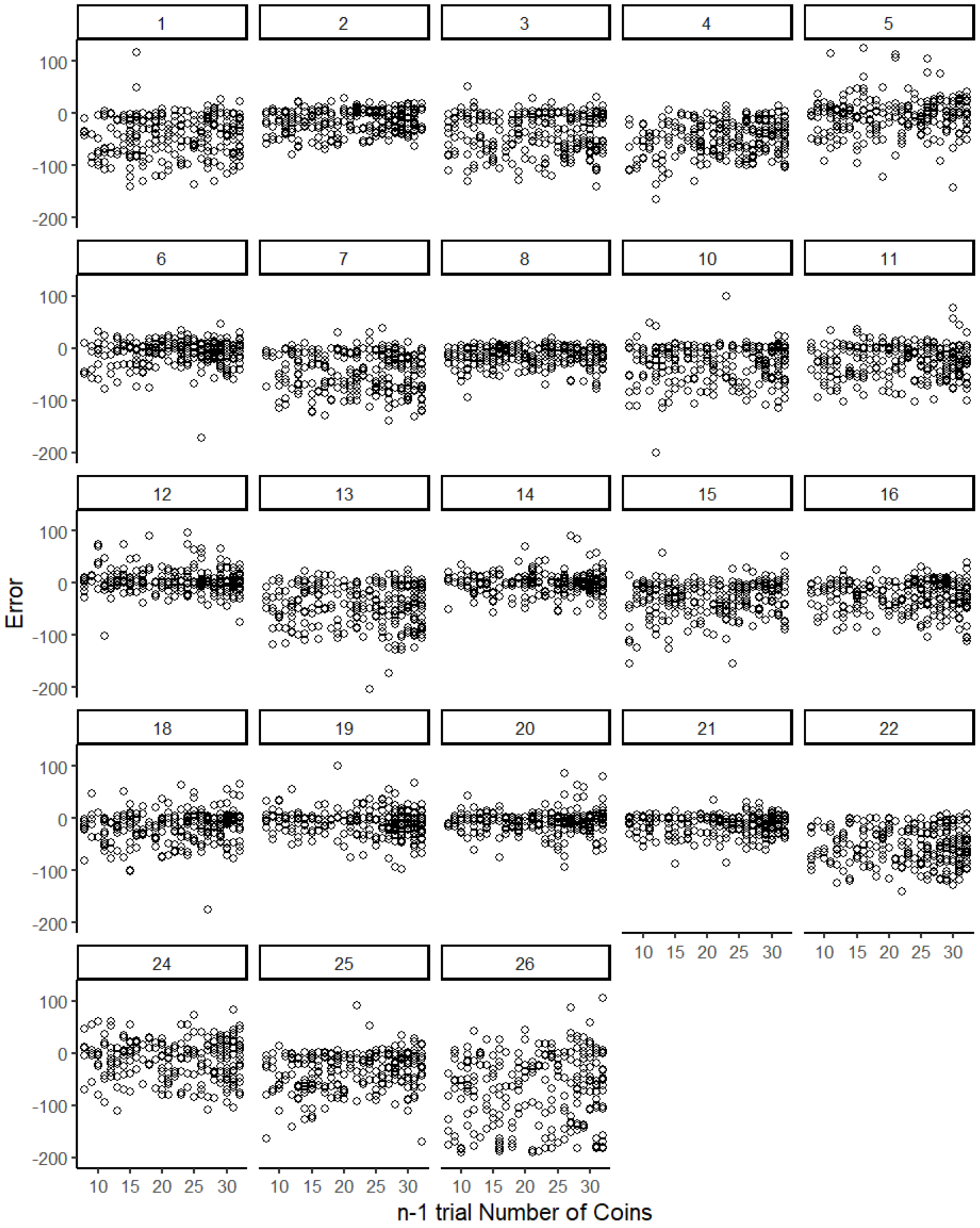

Figure S9. Distribution of errors as a function of the number of coins in trial n-1 in Experiment 2. The circles in the graph indicate the error value for each participant on each trial. The numbers in the rectangles represent the participant number.

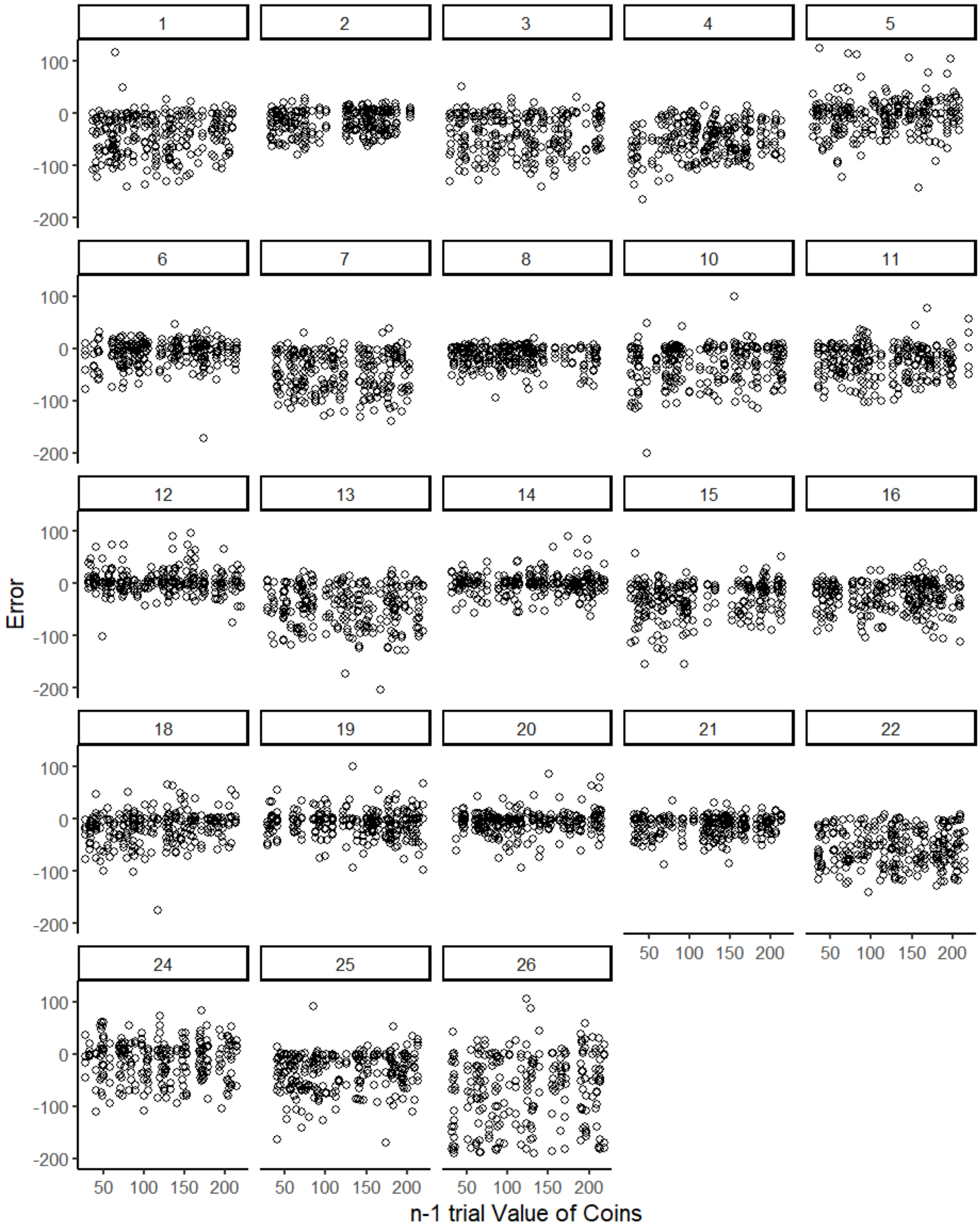

Figure S10. Distribution of errors as a function of coin value in trial n-1 in Experiment 2.
The circles in the graph indicate the error value for each participant on each trial. The numbers in the rectangles represent the participant number.

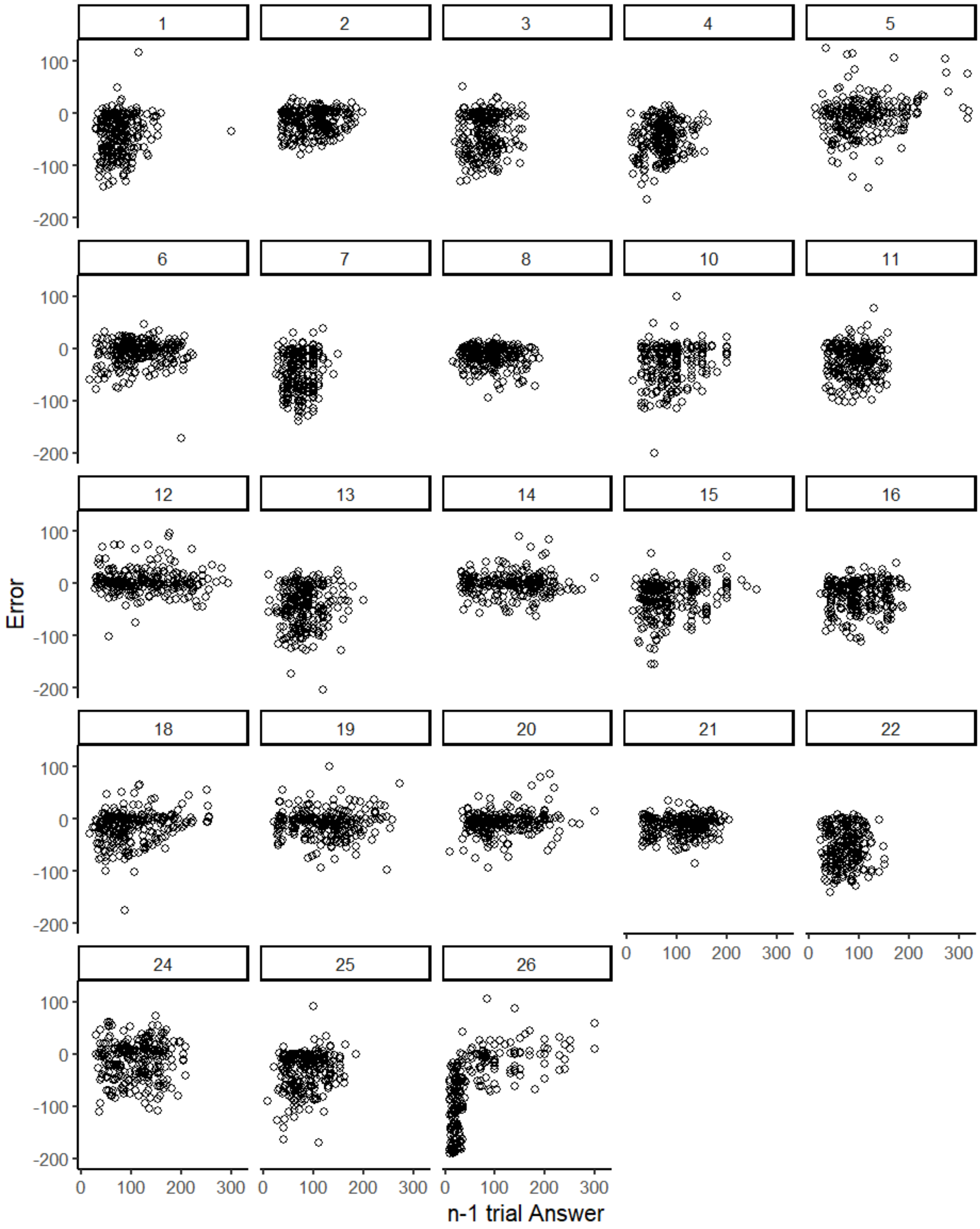

Figure S11. Distribution of errors as a function of the answer in trial n-1 in Experiment 2.
The circles in the graph indicate the error value for each participant on each trial. The numbers in the rectangles represent the participant number.

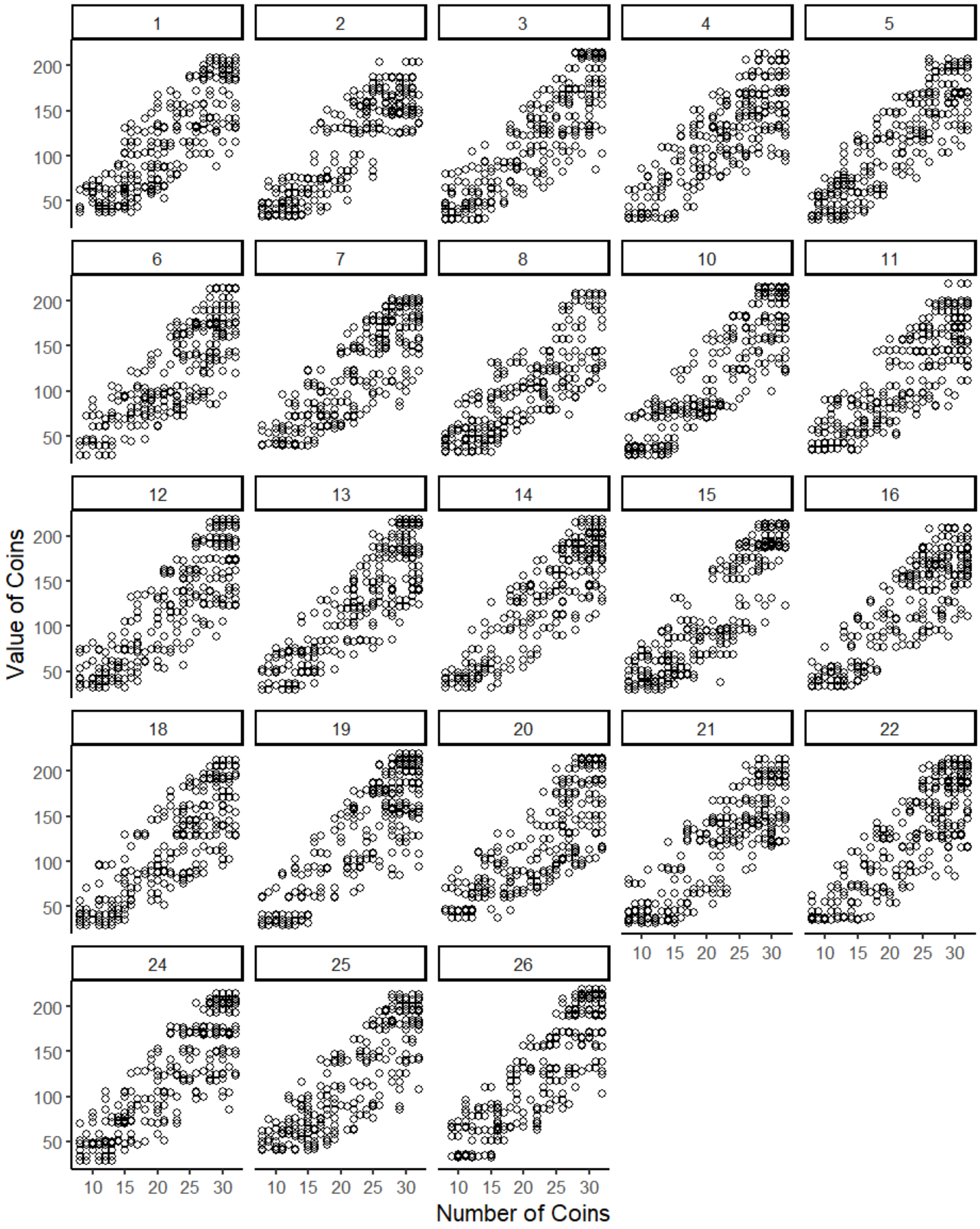

Figure S12. Distribution of the number of coins as a function of coin value in the current trial in Experiment 12.

The circles in the graph indicate the error value for each participant on each trial. The numbers in the rectangles represent the participant number.

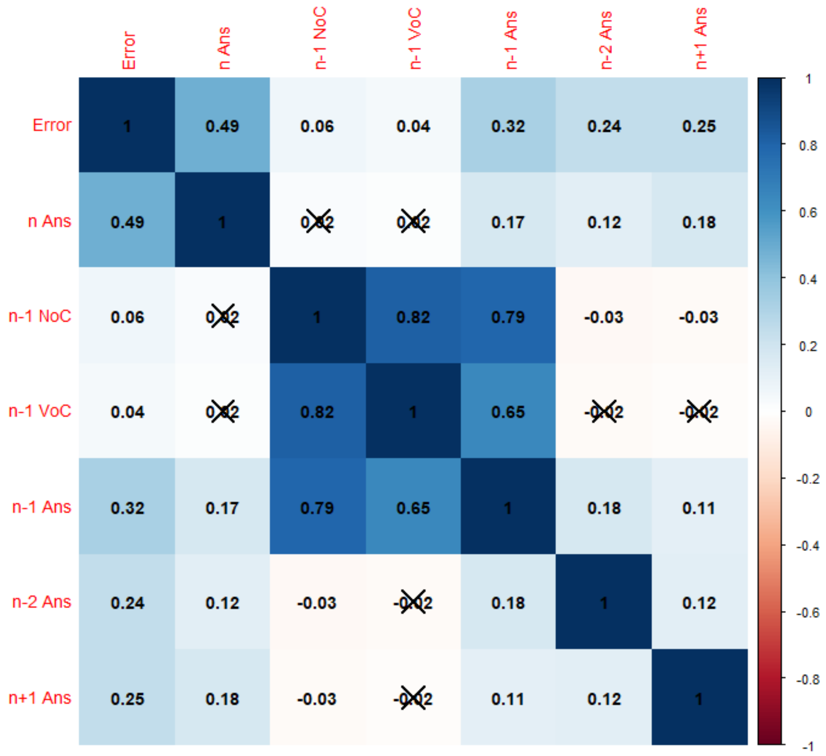

Figure S13. Correlation matrix of explanatory variables used in multiple regression analysis in Experiment 1. Cross marks indicate that the correlations are not significant. Error indicates the difference between the participant's answer and the actual number of coins.

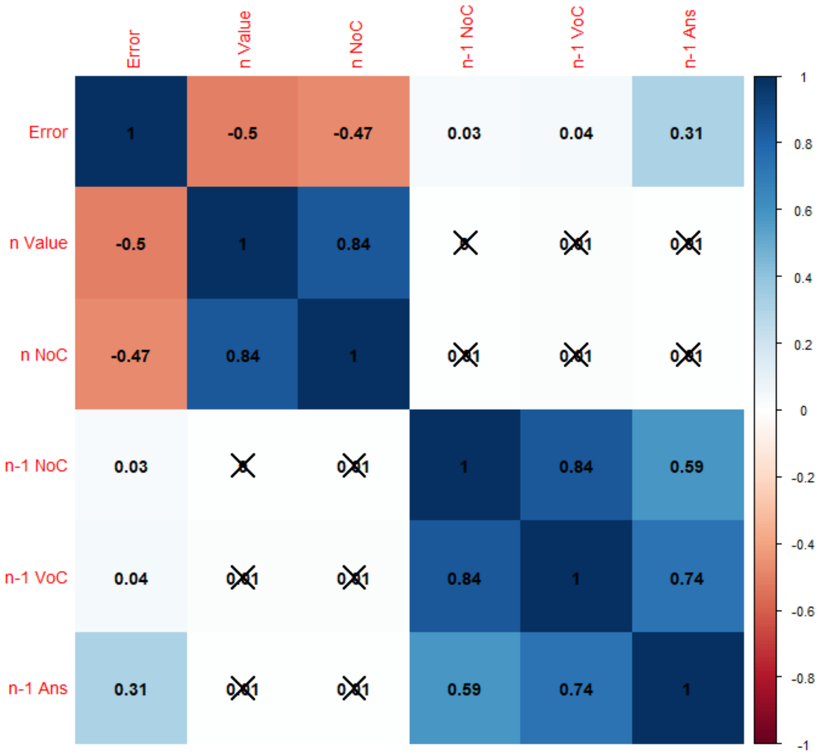

Figure S14. Correlation matrix of explanatory variables used in multiple regression analysis in

Experiment 2. Cross marks indicate that the correlations are not significant. Error indicates the difference between the participant's answer and the actual value of coins.

Ridge regression

To test whether problems caused by multicollinearity emerged in the multiple regression analysis, we performed ridge regression (Hoerl & Kennard, 1970) for each participant, which was performed by the glmnet package in R (Friedman et al., 2022).

Ridge regression requires a parameter λ to determine the influence of the penalty term: the higher the value of λ is, the greater the influence of the penalty term for regularization. λ was set for each participant. We randomly split each participant's data into five subsets and select the value of λ at which cross-validation would result in the smallest least squares error of the model. This procedure was repeated 100 times for each participant. The average of the 100 values of λ obtained thereby was used as the value of λ for each participant. Ridge regression was then performed on all data for each participant.

The average results of the ridge regression analyses for all participants in Experiments 1 and 2 are shown in Tables S1 and S2. Compared to the results of the multiple regression analysis presented in Tables 1 and 2 in the main text, the absolute values of the average effects were smaller in general, and the number of significant explanatory variables increased. However, the sign for all explanatory variables did not change. The results of the ridge regression were generally similar to those of the multiple regression analysis, suggesting that no serious problems due to multiple covariance occurred.

Table S1. Summary of multiple ridge regression analyses for Experiment 1.
NoC indicates the number of coins, VoC indicates the monetary value of coins, and Ans indicates the participant’s response.

Factor	**Average effect**	**(95% CI)**	**N**	***t* value**	***d***	***p* value**		**Mean adj.R^2**
n-1 NoC	-0.034	(0.028)	22	-2.60	-0.54	0.023	*	
n-1 VoC	-0.002	(0.002)	22	-2.51	-0.52	0.023	*	
n-1 Ans	0.107	(0.044)	22	5.05	1.05	0.000	***	0.213
n-2 Ans	0.014	(0.012)	22	2.42	0.50	0.024	*	
n Ans	0.112	(0.071)	22	3.26	0.68	0.008	**	
n+1 Ans	0.017	(0.012)	22	2.90	0.60	0.015	*	

Table S2. Summary of multiple ridge regression analyses for Experiment 2
NoC indicates the number of coins, VoC indicates the monetary value of coins, and Ans indicates the participant’s response.

Factor	**Average effect**	**(95% CI)**	**N**	***t* value**	***d***	***p* value**		**Mean adj.R^2**
n-1 VoC	-0.039	(0.026)	22	-3.09	-0.64	0.005	**	
n-1 Ans	0.127	(0.061)	22	4.28	0.89	0.000	***	0.466
n VoC	-0.230	(0.081)	22	-5.91	-1.23	0.000	***	
n NoC	-0.921	(0.222)	22	-8.60	-1.79	0.000	***	
